# Meta-path Based Prioritization of Functional Drug Actions with Multi-Level Biological Networks

**DOI:** 10.1038/s41598-019-41814-w

**Published:** 2019-04-02

**Authors:** Seyeol Yoon, Doheon Lee

**Affiliations:** 1 Department of Bio and Brain Engineering, KAIST, 291 Daehak-ro, Yuseong-gu, 34141 Daejeon, Republic of Korea; 2 Bio-Synergy Research Center, 291 Daehak-ro, Yuseong-gu, 34141 Daejeon, Republic of Korea

## Abstract

Functional drug actions refer to drug-affected GO terms. They aid in the investigation of drug effects that are therapeutic or adverse. Previous studies have utilized the linkage information between drugs and functions in molecular level biological networks. Since the current knowledge of molecular level mechanisms of biological functions is still limited, such previous studies were incomplete. We expected that the multi-level biological networks would allow us to more completely investigate the functional drug actions. We constructed multi-level biological networks with genes, GO terms, and diseases. Meta-paths were utilized to extract the features of each GO term. We trained 39 SVM models to prioritize the functional drug actions of the various 39 drugs. Through the multi-level networks, more functional drug actions were utilized for the 39 models and inferred by the models. Multi-level based features improved the performance of the models, and the average AUROC value in the cross-validation was 0.86. Moreover, 60% of the candidates were true.

## Introduction

*Drug actions* are defined here as single biological processes that are affected by drugs. Drugs act on biological systems and produce therapeutic effects. To describe them, terms, such as *pathways*, *mechanisms of actions*, and *modes of actions*, have been used. The pathways and the mechanisms of actions represent molecular sequences that are triggered by a drug, and the modes of action are brief descriptions of the drug actions^1^, while ‘drug actions’ is a more general term to describe how drugs act.

Drug actions can be categorized according to the biological levels where they occur: molecular drug action, phenotypic drug actions, and *functional drug actions*. Molecular drug actions are defined as drug actions on proteins. These are common in studies of drug actions: target proteins, molecular paths, transcription factors, and so on. Phenotypic drug actions are drug actions on diseases. They have been empirically, clinically, and experimentally observed, including the indications, side effects, symptoms, and so on. Functional drug actions are defined as drug actions on biological functions such as GO terms^2^ (gene ontology terms). Functional drug actions have also been studied. They can be observed via wet experiments or be inferred from molecular drug actions such as DEGs^3–6^ (differentially expressed genes). While no database provides information on the functional drug actions using standardized identifiers such as GO terms, the DrugBank database provides information about them in plain text. We extracted the biological terms in the textual descriptions. GO terms are almost one third of the terms. For example, the *apoptotic process*, *G2 phase*, *DNA replication*, etc. were extracted from the description about the drug actions of etoposide, an anti-tumour drug.

Identification of functional drug actions is beneficial for drug development because it supports the therapeutic hypothesis of a drug and aids in repurposing drugs for other diseases^7^. The therapeutic hypothesis refers to the hypothesis that a drug cures a specific disease. First, it provides biological interpretations that support the therapeutic hypothesis^3,8^. For example, vasodilation is the functional drug action of captopril, which is an anti-hypertensive drug, and it provides biological interpretation of how the drug attenuates high blood pressure more than its target protein, the ACE protein^9^. The second benefit is that a new therapeutic hypothesis can be inferred through functional drug actions. For example, Iorio *et al*.^10^ inferred that fasudil may be repurposed for several neurodegenerative disorders because fasudil-induced genes are enriched for cellular autophagy, which is applicable to neurodegenerative disorders. This utility is more notable when the target disease has no associated gene. We observed that 83.5% of phenotypes in the SIDER^11^ (Side Effect Resource) have no associated gene based on the CTD^12^ (The Comparative Toxicogenomics Database).

Even though the utilities of functional drug actions have been demonstrated in drug development, information about the functional drug actions in relevant databases is not only described in plain text without standardized identifiers such as GO terms but is also scarce and confined to their own purposes in the drug development. Such information in the DrugBank was produced by only two or more curators using PubMed, drug references, Food and Drug Administration (FDA) labels, etc.^13^ Actually, wet lab experiments with cells, tissue, or animal models are a clear way to identify functional drug actions; however, the costs are high. Therefore, dry computational experiments have been conducted to prioritize the candidates for functional drug actions based on various drug-related information, accumulated biological networks, and computational techniques. The prioritized candidates will help to collect functional drug actions that have been already identified and to select proper candidates for investigation in wet lab experiments.

Previous computational studies have utilized linkage information on biological networks between drugs and functions to distinguish between drug-affected functions, i.e., the functional drug actions from the other functions. This was based on *molecular level biological networks* that consist of molecule-to-molecule and molecule-to-function relations. Drug-related molecules were used for drugs to access the molecular level biological networks. In most previous studies on functional drug actions, the functions that were enriched by drug-related molecules have typically been considered as the functional drug actions. We briefly introduce two of them below. Sun *et al*.^4^ attempted to determine the functional drug actions that are related to signal transduction of a drug. They collected signal transduction proteins between drug-related proteins and drug-induced proteins via a random walk on the networks of PPIs (protein-protein interactions). Then, the GO terms, which were enriched by the signal transduction proteins, were considered as the functional drug actions of the signal transduction. Napolitano *et al*.^5^ attempted to identify the disease-specific functional drug actions. They utilized a set of drugs for which the indications are the same. The pathways that are enriched by all the DEGs of the drugs were considered to be the disease-specific functional drug actions.

However, the previous studies are incomplete. They depend on molecular level biological networks; however, the current understanding of molecular level mechanisms of biological functions is still limited. For example, two-thirds of GO terms do not yet have associated genes. These GO terms cannot be covered by the previous studies. To overcome this limitation, we constructed and utilized *multi-level biological networks* that contained functions, phenotypes, molecules, and the relations among them. Actually, biomedical information has accumulated not only on the molecular level but also on other levels. For example, the UMLS^14^ (Unified Medical Language System) provides various non-molecular entities: functions, symptoms, anatomies, diseases, etc. It also contains the relations between them; however, the amount of relations of function-to-function, function-to-phenotype, and phenotype-to-phenotype are insufficient. Therefore, we decided to use co-occurrence information about biological terms to supplement these relations. Co-occurrence information has gradually been used in bioinformatics to complement the relations between non-molecular entities, such as symptoms, diseases, functions, and drugs. Zhou *et al*.^15^ measured disease-disease similarity based on co-occurrences of symptom MeSH (Medical Subject Headings) terms, and they asserted that it was useful to infer the similarity. Brown *et al*.^16^ measured the drug-drug similarity based on the co-occurrence and concluded that it aided drug repurposing. Wang *et al*.^17^ gathered associations between drugs and genes based on their co-occurrence in the same sentence in an abstract or title. Himmelstein *et al*.^18^ gathered associations between diseases and tissues based on their co-occurrence frequency.

The expanded information on the multi-level biological networks decreased the number of missed functional drug actions. For the pairs of functional drug actions and the corresponding targets, the percentage of unconnected pairs decreased from 12% in molecular level networks to 7% in the multi-level networks. Such expanded information, however, may imply accuracy loss in the approach to functional drug actions. To mitigate this loss, we utilized linkage information between functions and drug *indications* as well as linkage information between functions and drug *targets*. This narrowed the scope of our approach to the functional drug actions. An indication refers to a phenotype that makes a drug advisable. Before the introduction of indications, we had to confirm that the indications had enough linkage information with the functional drug actions in the multi-level biological networks. We determined that for the pairs of functional drug actions and the corresponding indications, 54.0% of them were connected within the 2 shortest distances in the multi-level networks.

Of note, we intended to use the general terms of molecules and phenotypes. This is because, although we only used genes as the molecules, other kinds of drug-related molecules, such as DNA, epigenetic pattern, and protein complex, can be utilized in these studies. We also considered adding more phenotype types, such as symptom, observation, and appearance. Therefore, in this study, the targets are the genes for which proteins are bounded by a drug. The genes represent the molecules. The indications are the diseases that are cured by a drug. The diseases represent the phenotypes.

## Results

**Figure 1:**
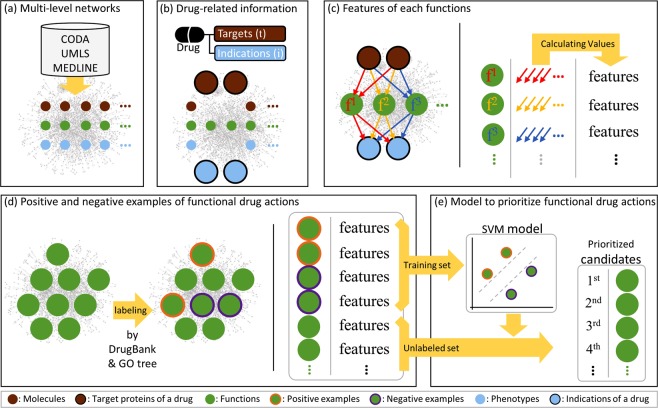
Method overview. (**a**) The multi-level biological networks were constructed from information stored in the CODA, the UMLS, and MEDLINE. (**b**) The target proteins and the indications of drugs were used for a drug to access the multi-level networks. The targets are the genes of which proteins are bounded by a drug. The genes are the molecules. The indications are the diseases that are cured by a drug. The diseases are the phenotypes. (**c**) Proper features of each function were required to distinguish the drug-affected functional concepts, i.e., the functional drug actions from the other functional concepts. The features were calculated from the paths between the targets and each function and from the paths between each function and indications. (**d**) We obtained the positive examples of the functional drug actions from the DrugBank. The negative examples were inferred from the positive examples and from Gene Ontology hierarchical structures. (**e**) The SVM model for a drug was build based on the examples and their features. The model estimated the possibility of drug actions on unclassified functions by prioritizing candidates of the functional drug actions.

This study consisted of five parts as summarized in Fig. 1. The first part was to construct multi-level biological networks. A given drug was replaced with its target proteins and indications to approach functions in the networks. The features of each function were extracted from linkage information about the function with targets or indications in the multi-level biological networks. The fourth part involved obtaining positive and negative examples of the functional drug actions for cross-validation. Finally, we built an SVM (Support Vector Machine) model based on the examples and their features. The model produced a ranked list of candidates for the functional drug actions of the drug.

### The multi-level biological networks

We defined three types of nodes: molecule, function, and phenotype. To define the types of relations between the nodes, we combined the relation types pre-defined in CODA and the original resources. The molecule type in this study consisted of proteins. Proteins take the main roles in drug actions as receptors and as transcription factors. The function type consisted of GO terms. The GO terms include the prevailing and well-organized ontology for functional concepts and include molecule functions, biological functions, and cellular components^2^. The phenotype consisted of diseases. We considered the functions and the phenotypes as single nodes in our networks. This is the same way a single protein has traditionally been considered as a single node in networks even though a single protein is a set of amino acids, structures, and domains.

For construction of the networks, we used information from published databases: CODA^19^, UMLS^14^, and MEDLINE^20^. The CODA (Context-Oriented Directed Association) is a multi-level biological database that we developed. Although the CODA provides enough relations that are molecule-to-molecule and molecule-to-function, it contains few relations between the functions and phenotypes. Therefore, we also used the UMLS and MEDLINE. The UMLS is a well-known multi-level biological database and is run by the NIH. There were too many pre-defined relation types in the UMLS to select or categorize them. We used only the original resources of relations to define the types of relations for this study. From MEDLINE, we introduced co-occurrence information from MeSH terms to reinforce the relations between the functions and phenotypes. As a result, the number of nodes in our networks was 82,439, which consisted of 20,707 molecules, 17,675 functions, and 44,057 phenotypes. The relations from CODA, UMLS, and MEDLINE are shown in Table 1. The relation types we defined are combinations of the database, the resource, and the relation type in the CODA. They are more explained in the methods section.

**Table 1:** The number of relations in the multi-level biological networks.

Type of relation			Type of node pair				
Database	Resource	Relation types in CODA	M → M	M → F	F → F	F → P	P → P
CODA	BioGRID	Undirected link	274,314	—	—	—	—
	RegNetwork	Directed link	18,226	—	—	—	—
	TRANSFAC	Directed link	14,116	—	—	—	—
	KEGG	Directed link	442	—	—	—	—
		Undirected link	51,696	—	—	—	—
		Positive increase	53,504	—	—	—	—
		Positive decrease	10,546	—	—	—	—
	EndoNet	Directed link	4,988	15	—	—	—
		Positive increase	214	388	—	—	—
		Positive decrease	355	4	—	—	—
		Negative increase	—	14	—	—	—
	GO	Undirected link	—	265, 115	—	—	—
	PhenoGO	Undirected link	—	—	20	6,678	—
UMLS	NCI	—	—	6,463	480	451	58,588
	OMIM	—	—	—	—	170	127,706
	MEDLINEPLUS	—	—	—	2	68	1,844
	MTHMST	—	—	—	—	—	330
MEDLINE	Co-occurrence	—	—	—	133,956	401,066	2,750,462
	**SUM**		**428,401**	**271,999**	**134,458**	**408,433**	**2,938,930**

This table shows the separate number of relations according to the type of relation and type of node pair. The relation types are defined by combination of the database, the resource, and the relation type in the CODA. The relations among molecules from the CODA were sufficient. The molecule-to-function relations seemed to be sufficient from the CODA and the UMLS. The number of relations between the functions and phenotypes from the CODA were not sufficient. Therefore, MEDLINE and the UMLS mainly provided the relations between the functions and phenotypes.

M: ^※^Molecule, F: Function, P: Phenotype.

### Drug-related information

Drug-related information was used for drugs to gain access to the biological networks. The linkage information between drug-related information and functions was utilized to discriminate between the functions that are affected by drugs, i.e., the functional drug actions, from other functions. We used target proteins and indications of the drugs.

Target proteins of the drugs were used as drug-related molecules in this study. They are typically used in studies of functional drug actions along with DEGs. Target proteins are essential entities in drug actions and trigger consequential molecular drug actions such as alterations of gene expression. From the DrugBank, we gathered target proteins of drugs when it was mentioned that their *Pharmacological Action* is known.

Indications of drugs were used as the drug-related phenotypes in this study. The phenotypic drug actions, such as indications, side effects, and symptoms, have been accumulated in several resources, including in CTD, OMIM^21^, and SIDER. To our knowledge, the phenotypic drug actions have not yet been used in studies of functional drug actions. From the CTD, we gathered indications of drugs when it was tagged as *Therapeutic*.

As a result, there were 1,311 drugs that had at least one target protein. Among them, the 428 drugs had at least one indication. One target was bounded by 3.03 ± 3.53 drugs, and one indication was treated with 4.76 ± 8.00 drugs. There were no drugs with the same targets and indications. The (±) sign means the standard deviation.

### Features of each function

We needed the features of each function to differentiate the functional drug actions from other functions. In this study, the features consisted of three values calculated from the linkage information, which them became all the meta-paths connecting a given function to the two kinds of drug-related information: targets or indications.

First, the linkage information was substantialized as the heterogeneous paths in the multi-level biological networks, as illustrated in Fig. 1c. For a given function, target-to-function paths and indication-to-function paths were extracted. There could be more than one target or indication.

**Figure 2:**
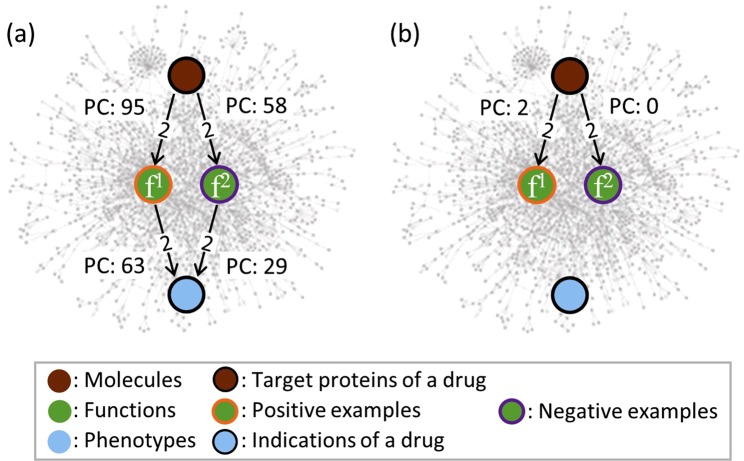
Comparison of a positive example and a negative example. (**a**) Enalapril is an anti-hypertensive drug. ACE is its target protein. Vasoconstriction (f1) is a positive example of the functional drug action of enalapril, whereas sex differentiation (f2) is a negative example. In the networks we constructed, the shortest distances were identical. The path count (PC) did not seem to be very distinguishable. (**b**) In a certain meta-path, there were two paths between ACE and the positive example, whereas there was no path between ACE and the negative one. This meta-path was composed of three node types: molecule, molecule, and function. The two edge types were the undirected link from the BioGRID in the CODA and the undirected link from the GO in the CODA. The targets are the genes for which the proteins are bounded by a drug. The genes are the molecules. The indications are the diseases that are cured by a drug. The diseases are the phenotypes.

The paths can provide us with a variety of features. However, the basic features, such as the shortest distance and the path count, is limited in this study because they have commonly been used for homogeneous paths but not heterogeneous paths. As shown in Fig. 2a for a small-scale analysis, it appeared that the shortest distances and the path counts were sometimes not good at distinguishing the functional drug actions from the other functions.

However, it appeared that the path counts in a type of paths were more useful for distinguishing the positive examples from the negative examples as shown in Fig. 2b. The types of paths are called *meta-paths*^22–25^. We determined to utilize the meta-paths to extract the features from the heterogeneous paths. A meta-path is a sequence of node types and edge types between two nodes at the abstract level^24,25^. Meta-paths have recently been used to investigate biomedical associations on heterogeneous biological networks. Fu *et al*.^23^ predicted the interactions between a drug and its target proteins. Himmelstein *et al*.^18,26^ predicted disease-associated genes and predicted compound-disease associations for drug repurposing. They analysed the known positive biological associations by meta-paths and attempted to determine the novel biological associations.

**Figure 3:**
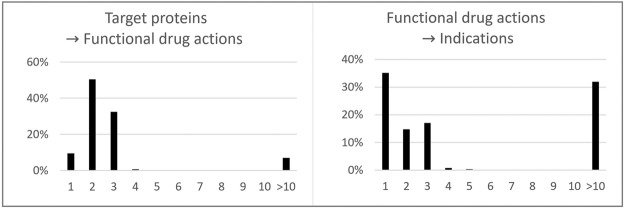
The distribution of the shortest distances of the two-node pairs. The x-axis indicates the shortest distance of pairs, and the y-axis shows the ratio of each bin. We measured how far the positive examples of the functional drug actions were located from the corresponding targets or indications. The results are shown in these graphs. Note that the direction was taken into account in this calculation.

We categorized all of the targets-to-function paths and the function-to-indications paths into meta-paths. The meta-paths are types of paths. In this study, the meta-paths were defined as follows. They could contain three node types: molecule, function, and phenotype. They could contain 18 edge types that are described in the methods section. Their maximum lengths were determined to be 3 with targets and 2 with indications. These lengths were decided on based on the distribution of the shortest distances between targets and the functional drug actions and those between the functional drug actions and indications (Fig. 3). Within a distance of ten or less, two-thirds of the functional drug actions are located within 3 and 2. We presented 20 examples of meta-paths for the enalapril case in the Supplementary Material (Supplementary Table S5).

**Figure 4:**
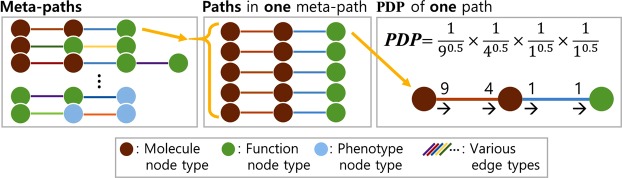
Method to calculate the PDP. The PDP was calculated by the in-degree and out-degree for all nodes in one path by raising each degree to the -w power, where w  ≥ 0 and is called the damping exponent, and by multiplying all exponential degrees to yield the PDP. The damping exponents were set to 0.5 in this study.

From one meta-path, we calculated three values: the path count, the sum of the PDPs (path-degree products), and the maximum PDP. The path count is the number of paths in a meta-path^22,23^. If the path counts were solely considered, the specificity of a path was ignored. Thus, the paths containing low-degree nodes could be underestimated, and the paths containing high-degree nodes could be overestimated. The path-degree product (PDP) has been used to measure the specificity of one path^18^. The PDP was calculated with the degrees of nodes in one path, as shown in Fig. 4. All three kinds of values from every meta-path constituted the features of each function.

Then, we selected some informative features from all the features because there were too many features in each drug case. In total, 3,732 meta-paths were possible to appear according to the above definition of node types, relation types and maximum lengths. Additionally, 11,196 features were possible to appear because each meta-path provides three values. In each of the 39 drug cases in the application section, the number of features decreased but remained over hundreds. To select the informative features for each drug case, we calculated the coefficients of linear regression for each feature with the positive and negative examples. For each drug case, we selected the top 20 features because the AUROC (Area Under the Receiver Operating Characteristic) value was higher for this case than when selecting a different number of top features.

### Examples of functional drug actions

We needed the functions that were labelled as functional drug actions. We called these labelled functions *positive examples* of the functional drug actions. The positive examples appear throughout this study, such as when measuring the shortest distances. They were also needed to train the SVM models in the application section.

Although no database provides positive examples with standardized identifiers, the DrugBank provides the textual descriptions of the drug actions in two data fields: *Pharmacodynamics* and *Mechanism of Action*. We extracted the GO terms from the descriptions via MetaMap^27^, which assigns UMLS concept IDs to biological terms. If possible, these concept IDs were converted into GO terms. The GO terms were expanded on the GO hierarchical structures.

We assumed that the negative examples were the GO terms with *zero* topology-based similarities^28^ to all of the positive examples in the GO hierarchical structures. The topology-based similarity of two nodes is *zero*, if they have only one common ancestor in a hierarchical structure. This process was conducted only if the number of positive examples was three or more in the same GO namespace. Of note, the multi-level biological networks we constructed are independent of the GO hierarchical structures.

As a result, among the 428 drugs with at least one target and one indication, all of them had at least one positive example. The numbers of positive examples for the drugs were 4.25 ± 3.58. We attempted to make this extraction procedure reproducible and objective; thus, we omitted the manual curation part. Additionally, the manual curation that we carefully performed in eleven of the drug cases showed that the accuracy of the extracted positive examples was acceptable; the precisions were 0.86 ± 0.08. The eleven drugs were selected in descending order of the number of positive examples among the 39 drugs in the application section. The results of the manual curation are presented in the Supplementary Material (Supplementary Table S1).

### Application

We applied our technique to 39 various drugs. In total, 428 drugs had at least one target, one indication, and one positive example. We observed that when there were more positive examples, the performance was better. We applied our technique to drugs with ten or more positive examples; 39 drugs remained of the 428 drugs. In the Supplementary Material (Supplementary Table S1), we present the positive examples for the 39 drugs. We also present the negative examples for the drugs in the Supplementary Material (Supplementary Table S2). They had various indications: tumours, hypertension, inflammatory disorders, local pain, vitamin D deficiency, etc. Following the technique shown in Fig. 1, we built 39 SVM models for each of the 39 drugs with their information: targets, indications, and the positive and negative examples of the functional drug actions. We utilized the targets-to-function meta-paths and the function-to-indications meta-paths in order to distinguish the positive and negative examples of the functional drug actions. In case of the enalapril case, 194 meta-paths appeared. And, we presented them in the Supplementary Material (Supplementary Table S3). Then, we constructed the 39 SVM models to prioritize candidates for the functional drug actions. We present the top 10 candidates of the 39 drug cases in the Supplementary Material (Supplementary Table S4).

Our technique of utilizing multi-level biological networks can cover more functional drug actions compared with previous methods that have utilized only molecular level biological network. We found that 19% of the positive examples had no associated gene. They could not be utilized in any of previous studies but could be utilized in this study. Then, 18% of the top 10 candidates had no associated gene. They could not be inferred in any previous studies but could be inferred in this study. This enlarged coverage was because of the multi-level biological networks that include functional and phenotypic relations.

**Figure 5:**
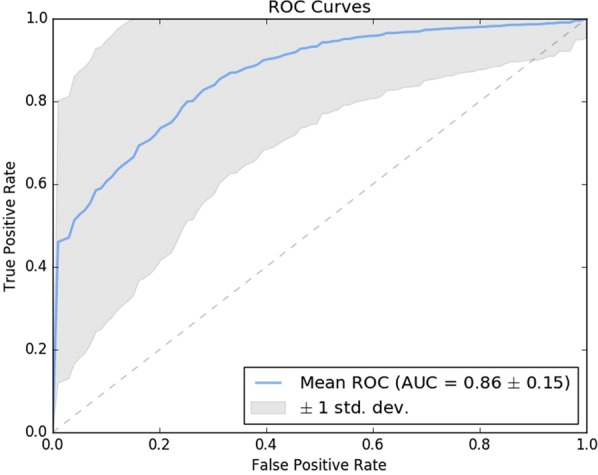
The ROC curves of the 39 drug cases. These were produced using four-fold cross validation over 20 iterations. The mean AUROC value was approximately 0.86, and the standard deviation was 0.15.

Each of the 39 models was able to be cross-validated because of the positive examples, and the AUROC value was 0.86 ± 0.15, as shown in Fig. 5. The AUROC values for 37 cases were above 0.70, showing a consistent performance. We could not compare our technique to previous techniques because none of the previous methods yielded an indicator of performance, such as AUROC values. They only mentioned the functional drug actions they predicted or suggested that they were consistent with previous literature or prior knowledge. They were all based on human decision and not objective validation.

However, the previous techniques can be simply viewed as approaches for determining functional drug actions via molecular level networks. Similarly, our technique is an approach for it via multi-level networks. The features used in this study can be grouped into those based only on molecular level networks and those related to multi-level networks. We confirmed the ratios of the two groups in the 20 selected features for the SVM model construction. The ratios could assess the relative usefulness of the two groups in the study of the functional drug actions. We determined that the 20 selected features had more of the features related to the multi-level networks than the features based only on the molecular level networks. The numbers of features based only on the molecular level networks were 7.44 ± 5.24 in the 39 drug cases. For the features related to the multi-level networks, there were 9.18 ± 4.52 features with function-to-function relations from targets to functions and 3.38 ± 2.37 features from functions to indications through the relations among functions and phenotypes. Multi-level-based features were more useful for the study of the functional drug actions compared with molecular level-based features. As an example, we presented the top 20 features for the enalapril case in the Supplementary Material (Supplementary Table S5).

We had the 39 SVM models prioritize candidates for the functional drug actions from the unlabelled functions. Within the top candidates, we looked through the literature to evaluate whether each candidate is the functional drug action. First, the drugs that were to be investigated were selected. We selected drugs from the 39 drugs if its AUROC value was higher than the average AUROC value, and we grouped them into five according to their indications. In each of the five groups, we selected one drug that had the most search results in Google Scholar, where we can search for even the most obscure information^29^. The five drugs included enalapril, paclitaxel, bupivacaine, calcitriol, and fluocinolone acetonide. For these five drugs, the top 10 candidates for the functional drug actions were manually evaluated. As a result, 60.0% were found to be actual functional drug actions of the corresponding drugs, as presented in the Supplementary Material (Supplementary Table S6). Moreover, most were not similar to the positive examples already provided in the DrugBank. They were expected to be novel functional drug actions not yet stored in the databases. We describe some of them with literature evidence below.

Enalapril is an anti-hypertensive drug. The top 10 had action potential (GO:0001508). Enalapril increases the action potential amplitude and the resting potential, and it has been suggested that the consequential rise in cardiac refractoriness may give anti-arrhythmic properties to enalapril^30^. Its functional drug action, action potential, provides a biological interpretation of how enalapril alleviates arrhythmia. Paclitaxel is an anticancer drug. Metaplastic ossification (GO:0036074) was included in the top 10. Metaplastic ossification is an abnormal formation of bone in normally soft structures. We found that paclitaxel and zoledronate synergistically reduced the incidence of bone metastasis from lung cancer and prolong survival^31^. Even though metaplastic ossification has no associated gene, it could be successfully prioritized in this study with multi-level networks. In the case of bupivacaine, which is a local anaesthetic drug, growth (GO:0040007) and locomotion (GO:0040011) were in the top 10 candidates. We found that bupivacaine inhibited cellular proliferation and migration in cancer cell lines^32^. Calcitriol is an active form of vitamin D. Cell death (GO:0008219), cell division (GO:0051301), mitotic cell cycle (GO:0007067), and endoplasmic reticulum (GO:0005783) were in the top 10 and are involved in the anticancer activities of calcitriol^33–36^. Fluocinolone acetonide is a glucocorticoid derivative used for skin disorders. Eating behaviour (GO:0042755), wound healing (GO:0042060), intestinal absorption (GO:0050892), and phototransduction (GO:0007602) were in the top 10. It has been reported in the literature^37–40^ that these four were affected by glucocorticoid, while no associations with fluocinolone acetonide were reported.

## Discussion

We showed that the introduction of multi-level biological networks is useful in the study of the functional drug actions. Compared with previous studies that depend only on molecular level biological networks, our technique utilized and inferred more functional drug actions. Moreover, multi-level network-based features were more useful in the prioritization than molecular level network-based features. In the bioinformatics domain, several studies^18,23,26^ have shown the utility of introducing multi-level networks. Currently, it is faster to utilize multi-level networks for various problems in bioinformatics.

For the first time, we cross-validated the predictive models for functional drug actions using positive examples of the functional drug actions. We confirmed that our technique had presentable and consistent performance for various 39 drugs. As more positive examples are collected, our technique could be applied to more drug cases. We expect that cross-validation will become common in the study of functional drug actions. In addition, there will be more databases that provide functional drug action information.

Many of the top candidates prioritized by our technique were found to be actual functional drug actions, as described above. When referring to the top candidates, it will be much easier to collect the functional drug actions that have already been experimentally identified in the literature but not yet stored in databases. In addition, the top candidates of which relevance is not found in the literature are still likely to be functional drug actions. Therefore, they may be promising subjects for wet lab experiments for drug actions. This pre-screening will reduce the cost of the discovery of functional drug actions.

## Methods

### The multi-level biological networks

The molecule type included genes, and the function type included GO terms. The phenotypic type included diseases that are MeSH terms in disease categories, and the concepts from some UMLS concept types: *Congenital Abnormality, Acquired Abnormality, Finding, Pathologic Function, Disease or Syndrome, Mental or Behavioural Dysfunction, Cell or Molecular Dysfunction, Sign or Symptom, Anatomical Abnormality*, and *Neoplastic Process*. Those UMLS concept types contained a considerable number of the MeSH terms in the disease categories.

Then, 18 types of biological relations were defined by combination of the database, the resource, and the relation type in the CODA. The 18 types are presented as the 18 rows in the Table 1.

From the CODA database, 13 types of relations for this study were defined by combining the six original resources and the eleven relation types that were pre-defined in the CODA. The six original resources included BioGRID^41^, RegNetwork^42^, TRANSFAC^43^, EndoNet^44^, KEGG^45^, GO, and PhenoGO^46^, which were released before 2017. The six pre-defined types in the CODA included: *Undirected Link, Directed Link, Positive Increase, Positive Decrease, Negative Increase*, and *Negative Decrease*. Moreover, their reversal types were also made. *Undirected Link* means a non-directional association, and *Positive Decrease* means that the activity or the amount of a receiver decreases as the activity or the amount of an actor increases^19^.

From the UMLS database (version 2016AA), four types of relations were defined for the present study. They were defined only by their original resources: MedlinePlus^47^, MTHMST^48^, NCI^49^, and OMIM. Although the UMLS has more than 200 resources, only these four resources provided a considerable number of relations among the nodes that we considered. The pre-defined types of relations in the four resources were too numerous for us to properly categorize them. Therefore, we ignored them when defining the types of relations for this study.

MEDLINE database (version 2017) provided the co-occurrence frequency of the two MeSH terms. It provides how many times two MeSH terms have been attached to the same biomedical literature reference. Only one type of relation was defined for our research. Among the functions or phenotypes, if two nodes had any co-occurrence frequency, we determined that there is a co-occurrence relation between them. We attempted to separate them according to their frequency. However, the performance (AUROC values) in the application was best when the co-occurrence relations were not separated.

### Software implementation

We wrote most of the code in Python language to implement tasks, such as building networks, extracting paths, and scoring meta-paths. We utilized common libraries, such as *sklearn*, *numpy*, and *matplotlib*, to select the top 20 features based on linear regression coefficients, to build the SVM models, and to show the results. We also utilized the *dagofun* library to infer the negative examples of the functional drug actions based on the positive examples.

## Supplementary information

Table S1. The positive examples of the functional drug actions.

Table S2. The negative examples of the functional drug actions.

Table S3. The meta-paths of the enalapril case.

Table S4. The top 10 candidates for the functional drug actions.

Table S5. The top 20 features for the enalapril case.

Table S6. The top 10 candidates for the functional drug actions with evidence.

## Data Availability

A file containing the multi-level biological networks is available at 10.5281/zenodo.2530389. The main source codes and their main related files are available at 10.5281/zenodo.2579579.
